# The Effects of Iodine Fortified Milk on the Iodine Status of Lactating Mothers and Infants in an Area with a Successful Salt Iodization Program: A Randomized Controlled Trial

**DOI:** 10.3390/nu9020180

**Published:** 2017-02-22

**Authors:** Pantea Nazeri, Parvin Mirmiran, Zhale Tahmasebinejad, Mehdi Hedayati, Hossein Delshad, Fereidoun Azizi

**Affiliations:** 1 Nutrition and Endocrine Research Center, Research Institute for Endocrine Sciences, Shahid Beheshti University of Medical Sciences, 19395-4763 Tehran, Iran; nazeri.pantea@gmail.com (P.N.); jtahmasebinejad@yahoo.com (Z.T.); 2 Department of Nutrition and Clinical Dietetics, Faculty of Nutrition Sciences and Food Technology, National Nutrition and Food Technology Research Institute, Shahid Beheshti University of Medical Sciences, 19395-4741 Tehran, Iran; 3 Cellular and Molecular Research Center, Research Institute for Endocrine Sciences, Shahid Beheshti University of Medical Sciences, 19395-4763 Tehran, Iran; hedayati47@yahoo.com; 4 Endocrine Research Center, Research Institute for Endocrine Sciences, Shahid Beheshti University of Medical Sciences, 19395-4763 Tehran, Iran; delshad1336@yahoo.com

**Keywords:** iodine status, lactating mothers, infants, iodine fortified milk, urinary iodine, breast milk iodine

## Abstract

Iodine deficiency during the first two years of life may cause irreversible brain damage and mental retardation. The aim of the present study was to investigate, for the first time, the effect of iodine fortified milk on the iodine status of lactating mothers and their infants. In this multicenter randomized controlled trial, 84 lactating mother-infant pairs from health care centers were randomly selected. After meeting the inclusion criteria, lactating mothers were randomly assigned to two groups: the iodine fortified milk group and the control group (*n* = 42 each). Maternal and infant urine and breast milk samples were collected at 3–5 (baseline), 7, 10, 14 days, and 1 month postpartum, for a measurement of the iodine concentration. A total of 84 lactating mothers, with a mean age of 28.2 ± 4.5 years, and 84 infants, with a mean age of 4.2 ± 0.7 days, were included in the study. Compared to mothers of the control group, mothers receiving iodine fortified milk had higher urinary (*p* < 0.001) and breast milk (*p* < 0.001) iodine concentrations. Urinary iodine levels in infants revealed no significant differences between the two groups. The findings of this study indicate that supplementation with daily iodine fortified milk provides iodine nutrition adequacy among lactating mothers. However, it had no effect on the iodine status of infants, who were previously iodine sufficient.

## 1. Introduction

Iodine, as an essential component of thyroid hormones, is vital for normal growth and the development of most organs, especially the brain [1,2]; hence, iodine deficiency during the first few years of life can lead to developmental delays, irreversible brain damage, and mental retardation [3,4,5,6]. Based on the United Nations Children’s Fund (UNICEF) estimation, over thirty five million newborns currently remain unprotected against the lifelong consequences of brain damage associated with iodine deficiency [7].

It is now well recognized that iodized salt is the best way of guaranteeing an adequate amount of iodine in the diet, and this is the most effective approach for the control of iodine deficiency disorders (IDD) in almost all countries worldwide [8,9]; however, concerted efforts are currently being made in many countries to reduce salt intake, for the prevention of cardiovascular diseases and hypertension, which raises the concern that decreasing salt consumption will increase the risk of iodine deficiency [10,11,12,13]. In addition, it appears that, in some countries/areas, iodine requirements of the most susceptible groups, including pregnant and lactating women, might not always be adequately met by iodized salt [14,15,16]. On the other hand, as seen in most industrial countries, where only a part of the population actually consumes iodized salt, the increase in the urinary iodine concentration (UIC) of the population mainly depends on the presence of iodine in foods, viz. milk and dairy products. It has been shown that in several countries, such as the USA, New Zealand, Australia, Canada, Denmark, Belgium, Norway, and Germany, milk is a good vehicle for an adequate iodine intake [17,18,19,20,21,22,23,24]. Surveys on individual consumption reveal that the frequency of milk consumption is associated in a dose dependent manner with their urinary iodine concentrations [25,26]. 

In the Islamic Republic of Iran, which was recognized as an area of iodine deficiency, the production and nationwide consumption of iodized salt containing 20–40 ppm iodine began in 1990, and became mandatory for household consumption by 1994. Since then, national surveys conducted every 3–5 years have shown a sustainable elimination of iodine deficiency disorders among schoolchildren. Although Iran was declared to be free of iodine deficiency in the year 2000 [27,28], decreasing the intake of iodized salt as the main dietary iodine source has been accompanied by an increased percentage of subjects with insufficient iodine status [12], and hence, considering dietary iodine sources other than iodized salt is essential for vulnerable groups, specifically lactating mothers with higher iodine requirements. Moreover, despite the recommendations of major societies i.e., the American Thyroid Association and the Endocrine Society [29,30,31], the need for iodine supplementation in lactating mothers in Iran with a sustainable elimination of IDD over the past two decades, has not been yet evaluated. Therefore, the present study was designed to investigate the effect of iodine fortified milk on the provision of iodine adequacy among lactating mothers and their infants, in an area with a successful salt iodization program.

## 2. Materials and Methods

### 2.1. Subjects

In this multicenter randomized controlled trial, conducted in the southern region of Tehran, four health care centers responsible for newborn screenings were randomly selected. In each health care center, on the first visit and at the time of screening for congenital hypothyroidism (within 3–5 days postpartum), 20 lactating mothers and their infants were enrolled for participation in this study, if they met the following inclusion criteria: Healthy mothers and their infants had no history of thyroid disorders, were not currently using iodine containing supplements and disinfectants, had had a singleton birth and intended to exclusively breast feed; infants were born full-term (gestational age, 37–42 weeks) with an age of 3–5 days and a normal birth weight (2500–4200 g), and at that point in time (3–5 days),had normal serum thyrotropin in the neonatal screening program. Maternal information on the age, education, occupation, date of last pregnancy, gravidity, parity, history of abortion in previous pregnancies, use of iodine-containing supplements during pregnancy, and the type of delivery were documented, and demographic information on the newborn, including, birth of date, sex, and birth weight, height, and head circumference measurements, was obtained using an interviewer-administrated questionnaire. Written informed consent was obtained after the study protocol and objectives were fully explained to all lactating mothers and/or their husbands. The present study was approved by the ethics committee of the Research Institute for Endocrine Sciences (RIES), Shahid Beheshti University of Medical Sciences. This clinical trial has been registered in the Iranian registry of Clinical Trials at http://www.irct.ir with the following identification: IRCT20135074794N9.

### 2.2. Intervention

After meeting the inclusion criteria, lactating mothers were randomly assigned to one of two groups: iodized salt as the control group (*n* = 42) and the iodine fortified milk (*n* = 42) group, using a random number table. This randomization was conducted by a researcher who was not directly involved in the data collection. For an equal allocation to the two groups, the direction for reading the table was predetermined as the right and an arbitrary starting point was selected. Then, the researcher equated the odd and even numbers to the intervention and control groups, respectively. If mothers were lactose tolerant, they were assigned to the control group. In the control and iodine fortified milk groups, mothers received recommendations to use only iodized salt during cooking and as table salt. In the iodine fortified milk group, each mother received a box containing 15 tetra pack packets of sterilized milk every two weeks and was instructed to consume a packet of milk daily, with breakfast or the morning snack; 200 mL of which provided 150 µg iodine/day. Mothers were also asked to drink the milk directly, without heating it, since the process of heating may alter the iodine content of iodine fortified milk. All participants were telephoned weekly to ensure that the iodized salt and/or iodine fortified milk were consumed. The intervention in this study was started at the sixth day postpartum and lasted for four weeks. Compliance was evaluated by counting the empty packets and weekly follow-up phone calls.

To provide iodine fortified milk, organic cows’ milk was fortified by supplementing cattle feed with potassium iodide as the source of iodine [32,33]. Milk was sterilized for six months of its shelf life and then packaged into 200 mL portion packets. Ten tetra pack packets of sterilized milk were randomly chosen from different boxes and tested for quality control by the iodine laboratory of the Endocrine Research Center (ERC), the reference laboratory of the Eastern Mediterranean region. All packets of milk contained a mean iodine content (range) of 155 (151–160) µg/200 mL.

### 2.3. Urine and Milk Samples Collection

At the first visit, labeled plastic bottles and adhesive pediatric urine bags (SUPA medical services, Tehran, Iran) were provided, to collect spot urine samples of each lactating mother and their infant at 3–5 (baseline), 7, 10, 14 days, and 1 month postpartum, at any time during the day, according to the detailed instructions provided; they were also instructed to clean the genital region of the newborns and to place the entire penis in the bag, attaching the adhesive to the skin for boys and to fit the bag over the labia for girls. If urine samples of newborns could not be collected using an adhesive urine bag after three attempts, mothers were asked to do this by holding a specimen bottle in the urine stream. Mothers were also asked to manually express their breast milk at the same time points as these urine collections. At each time point, all urine and milk samples were collected and sent to the iodine laboratory of the ERC, and were transferred in screw-top labeled plastic vials. The aliquots were kept frozen at −20 °C, until the iodine concentrations were measured.

### 2.4. Salt Sample Collection

Two tablespoon salt samples, used during cooking and/or as table salt, were collected from each of the mothers at 3–5 days (baseline), and 1 month postpartum; some mothers used two types of salt (i.e., iodized salt and sea salt, or iodized salt and rock salt), for which samples of both were collected. The samples were kept in lightproof, closed plastic cans and labeled with the code for each mother.

### 2.5. Laboratory Measurements

The iodine concentration in urine and milk samples was analyzed using the Sandell-Kolthoff (acid-digestion) reaction [34,35] and results were expressed as micrograms of iodine per liter of urine and milk. Milk samples were carefully homogenized before the alkaline ashing procedure. Intra-assay coefficients of variation (CV) at UIC values of 8.5, 17.5, and 36.0 µg/L, were 8.5%, 6.2%, and 8.0%, respectively. The inter-assay CV at concentrations of 8.5, 17.4, and 36.4 µg/L, were 10.3%, 9.7%, and 8.0%, respectively. The intra-assay CV at breast milk iodine concentration (BMIC) values of 3.5, 12.7, and 36.2 µg/L, were 8.6%, 6.7%, and 9.3%, respectively. The inter-assay CV at concentrations of 3.3, 12.9, and 35.7 µg/L, were 9.8%, 8.6%, and 12.3%, respectively. The iodine concentration of salt samples was determined using the iodometric titration method, with 1 ppm sensitivity and 1% CV. The obtained values are shown in ppm. 

### 2.6. Definition

In lactating women and infants, according to international criteria, a median UIC <100 and ≥100 µg/L, is representative of deficient and sufficient urinary iodine, respectively [8]. Adequate BMIC was considered to be 150–180 µg/L [36]. 

### 2.7. Statistics Analysis

Based on our previous data on the median UIC in Tehranian women of childbearing age [12], to detect the change in the median UIC of 35 µg/L using a standard deviation (SD) of 55 µg/L with 90% power and a 2-side alpha = 0.05, and taking into account 30% attrition, at least 40 lactating mothers and their infants would be needed to be recruited into each of the iodized salt and iodine fortified milk groups.

The frequency distribution (percentage), mean ± SD, and median (interquartile range (IQR)) were expressed according to categorical and continuous variables. Normality of the variables was assessed by the Kolmogorov-Smirnove test and a histogram chart. Chi-square and Mann-Whitney ort-tests were used to assess the significance of differences for categorical and continuous variables in lactating mothers and infants. Linear mixed model analysis was used to assess the effect of iodine fortified milk on maternal and infant urinary and breast milk iodine concentrations. Three skewed outcome variables were log-transformed before analysis. For each continuous outcome variable, a separate mixed model was derived with the time and group as fixed effects, and participants as the random effect. Factors considered in the maternal UIC analysis were: Maternal UIC at baseline, mothers’ job, gravidity, use of iodine containing supplements, and iodine content of salt. For the infant UIC analysis, besides the five maternal factors, an additional three factors (i.e., birth weight, infant UIC at baseline, and use of formula during the study period) were also considered. Statistical analyses were completed using IBM SPSS for windows (version 20.0, 2011, IBM Corp., Armonk, NY, USA) and the R-3.0.2 statistical programming environment using the nlme package, with *p* values < 0.05 being considered as significant.

## 3. Results

**Figure 1:**
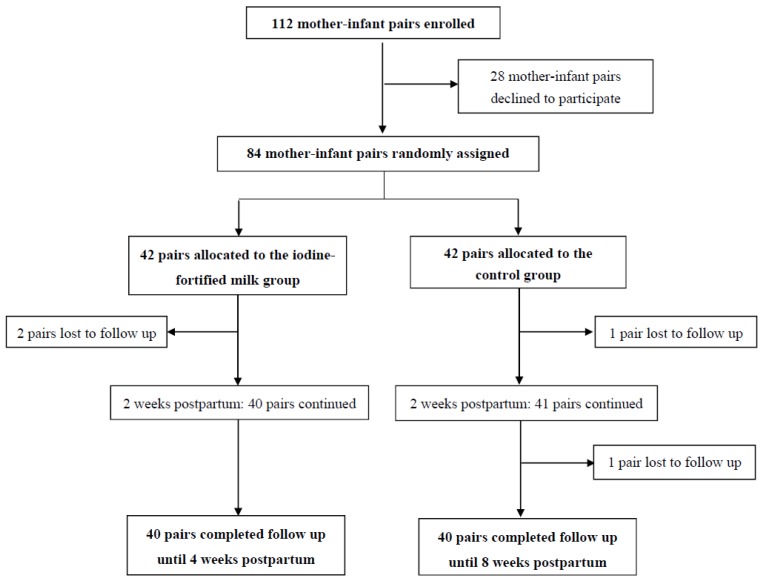
The study profile at a glance.

The flow of participants through the study is shown in Figure 1. Of a total 112 mother-infant pairs who initially enrolled, 28 mother-infant pairs declined to participate. Out of the eligible mothers who were willing to collaborate in this study, only three participants were lactose intolerant. A total of 84 mother-infant pairs were randomly allocated to the iodine fortified milk group and control group, (*n* = 42 each). Two weeks after supplementation, two mother-infant pairs were excluded from the intervention group, due to severe infant reflux and refusing to participate in the study; in the control group, one mother-infant pair was excluded, due to infant icter and hospitalization. Both study groups were followed for the following two weeks, during which one mother-infant pair was excluded from the control group; eventually, the data of 80 mother-infant pairs was made available for the current analysis (*n* = 40 each).The compliance rate was 94.7% in the intervention group.

At the baseline, the mean ± SD age of the mothers and infants were 28.2 ± 4.5 years and 4.2 ± 0.7 days, respectively. There were no significant differences in the maternal and infant baseline characteristics of the two groups, with regard to the following factors: Maternal age, education, date of last pregnancy, gravidity, parity, type of delivery, history of abortion, use of iodine containing supplements during pregnancy and neonatal sex, birth weight, height, head circumference, and thyrotropin concentration (Table 1). When focusing on the salt samples collected during the study period, the median iodine content ranged from 25.0 at the baseline, to 25.8 ppm at one month postpartum. No significant differences were found in the iodine content of salt between the two groups at the baseline and follow-up.

The BMIC and UIC of the mothers and infants, and the proportions of mothers and infants with an UIC ≥100, and a BMIC ≥150 µg/L, in the iodine fortified milk and control groups during the period of the study, are shown in Table 2. Maternal and infant median (IQR) UICs at 3–5 days (baseline) were 70.2 (41.2–199.0) and 231.2 (91.7–268.2) µg/L in the iodine fortified milk group and 96.9 (47.1–193.8) and 192.8 (79.4–244.6) µg/L in the control group, respectively. On the basis of their median UIC at the baseline, 35.9% and 45.7% of mothers, and 72.2% and 61.5% of infants, in intervention and control groups had an UIC ≥100 µg/L, respectively. No significant differences in maternal and infant UICs at the baseline were found between the two groups. The median (IQR) BMIC was 176.0 (133.7–218.7) µg/L in the iodine fortified milk group and 215.0 (168.5–315.5) µg/L in the control group, indicating statistically significant differences between the two groups (*p* = 0.027). At 3–5 days postpartum, the percentage of BMIC ≥150 µg/L was found in 65.0% and 81.0% of mothers of the intervention and control groups, respectively.

**Figure 2:**
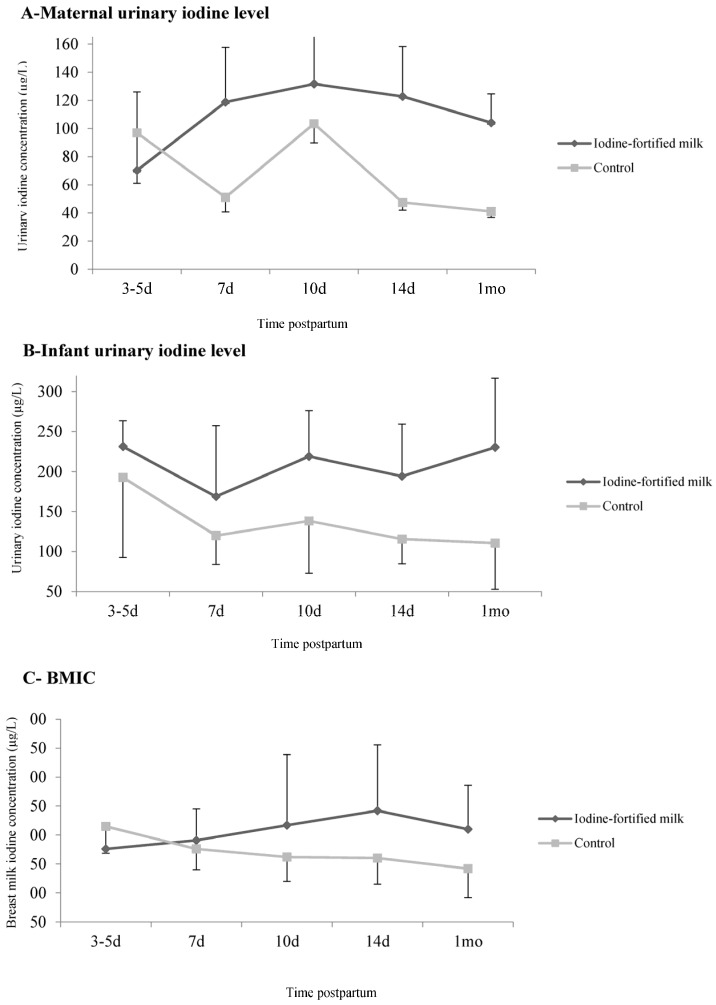
The effect of iodine fortified milk on maternal and infant urinary and breast milk iodine concentrations. (**A**) Urinary iodine concentration (μg/L) of mothers; (**B**) urinary iodine concentration (μg/L) of infants and (**C**) breast milk iodine concentration (μg/L). Data are presented as the median and error bars are the differences between the median, and the 1st and 3rd quartiles.

At the follow-up, the median (IQR) UIC of mothers in the intervention group increased from 70.2 (41.2–199.0) μg/L at the baseline, to 104.1 (57.3–160.9) μg/L at one month, although this was not significant; however, this value significantly decreased in mothers of the control group, from 96.9 (47.1–193.8) μg/L at the baseline, to 41.1 (32.1–55.9) μg/L at one month (*p* < 0.001). The median (IQR) maternal UIC in the control group remained below the 100 µg/L cutoff for iodine deficiency at all time points, except at 10 days postpartum (Figure 2A). Infants of mothers receiving iodine fortified milk had a median UIC ranging from 231.2 (91.7–268.2) to 230.2 (71.0–317.5) μg/L, demonstrating no significant difference over the intervention period, i.e., between three and five days, and one month postpartum (*p* = 0.339). However, the median (IQR) UIC decreased in the infants of mothers assigned to the control group, ranging from 192.8 (79.4–244.6) to 110.4 (47.2–197.0) μg/L throughout the study’s duration (*p* = 0.097) (Figure 2B). The median (IQR) BMIC gradually increased from 176.0 (133.7–218.7) μg/L at the baseline, to 210.0 (100.0–286.0) μg/L at one month, in mothers who received iodine fortified milk (*p* = 0.107); this value ranged from 215.0 (168.5–315.5) μg/L at the baseline, to 142.0 (92.2–197.2) μg/L at one month, in mothers of the control group (*p* < 0.001) (Figure 2C).

The effects of iodine fortified milk on maternal and infant UIC and BMIC, using the mixed-model analysis, are presented in Table 3. In lactating mothers who received iodine fortified milk, the UIC values improved significantly, when compared to the control group (*p* < 0.001). After further adjustment of the baseline maternal UIC, differences between the two groups remained significant (*p* < 0.001). Infant UICs increased significantly in the intervention group, compared to the control group; however, after an adjustment of the baseline infant UICs, no significant difference was found between the two groups over the course of the study. In mothers supplemented with iodine fortified milk, there was no significant increase in the BMIC values, when compared with the control group (*p* = 0.096); however, after further adjustment of the baseline values, significant increases were observed in the BMIC of mothers in the iodine fortified milk group (*p* < 0.001).

## 4. Discussion

To the best of our knowledge, this is the first study assessing the effect of iodine fortified milk on the iodine status of lactating mothers and infants in an area with a successful salt iodization program. Findings of the current study indicate that the supplementation of lactating mothers with iodine fortified milk improved maternal urinary iodine to an adequate status. The breast milk iodine concentration in mothers receiving iodine fortified milk was significantly higher than in those of the control group. In addition, the mean urinary iodine level of previously iodine sufficient infants, whose mothers received iodine fortified milk, did not show any significant differences, when compared to the control infants. 

The importance of milk and dairy products for the provision of adequate iodine is well-known; however, less information is available concerning the iodine content of milk, which meets the daily requirements of iodine in different age groups. Several studies have shown that the iodine concentration in milk varies between different countries and has changed significantly over the years. For instance, in Boston, the mean iodine concentration in milk was reported to be 45.4 µg/100 mL [22]. In England, the median iodine content was 14.5 µg/100 g in organic milk and 25.0 µg/100 g in non-organic milk [37], while data from Spain indicated a mean content of 25.9 µg/100 mL [26], and in Italy, a median value of 26.4 µg/100 mL was reported [38]. Data from Germany showed an increasing trend from 2004 to 2010 for the iodine content in milk; likewise, in Spain, the iodine content increased between 1991 and 2008 [39]. 

In Australia, the amount of iodine in milk reduced from 59.3 µg/mL in 1975, to 19.5 µg/mL in 2004, which can be explained by chlorine-containing sanitizers replacing iodine-containing sanitizers in the dairy industry, resulting in a lower iodine content of milk [21]. The United Kingdom (UK) never introduced a formal iodization program to ensure optimal iodine status, but increased advertising and promotion, and a rise in the consumption of organic milk resulted in an exacerbation of the mild iodine deficiency in the country [37,40]. A recent study conducted in Shahrekord, in central Iran, showed that the mean iodine content of milk in semi- and industrial dairy farms was 14.8 and 31.4 µg/100 mL, respectively [41]; however, due to a low daily per capita consumption of milk (<half of the recommended), it is not yet considered a main dietary iodine source among Iranian populations.

In countries where milk and dairy products are the main sources of dietary iodine, a higher milk iodine concentration and increased milk consumption have been cited as the reasons for the eradication of iodine deficiency. A strong association has been found between milk intake and urinary iodine excretion in several cross-sectional and follow-up studies [23,42,43]. Since milk is an appropriate source of iodine in children, findings of studies conducted in schoolchildren have demonstrated that those who consumed milk more often had higher urinary iodine concentrations [26]. However, few studies have addressed the role of cow milk as a source of iodine during pregnancy and/or lactation [44,45,46]. Moreover, there is an emerging concern during pregnancy and lactation, when a woman needs additional iodine in order to have adequate supplies for both her own and her baby’s needs. In the UK, organic milk has a significantly lower iodine concentration, compared to non-organic milk, and pregnant women who switch to organic milk are likely to have a reduced iodine intake [37,40]. Similarly, the risk of iodine deficiency has been observed among pregnant and childbearing aged women in the USA and Iceland, who do not consume milk and dairy products [46,47]. The present study indicates that lactating mothers who received iodine fortified milk containing 77.5 µg iodine/100 mL daily, had higher urinary and breast milk iodine concentrations, compared to controls.

Based on the current evidence, it appears that, in some countries, the iodine requirements of the most vulnerable groups, i.e., pregnant and lactating women, and children aged 6–24 months, are not always adequately met by iodized salt; to address this issue, in 2007, WHO/ICCIDD/UNICEF issued a joint statement on “*Reaching Optimal Iodine Nutrition in Pregnant and Lactating Women and Young Children*”, recommending that, besides strengthening universal salt iodization programs, additional complementary strategies such as iodine supplements, should be considered to ensure optimal iodine nutrition for these groups [48]. Moreover, the American Thyroid Association and the Endocrine Society [30,31] recommend that pregnant and lactating women take 150 µg iodine in daily prenatal vitamin/mineral supplements. The median urinary iodine at the baseline of the current study, in both the iodine fortified milk and control groups, also denoted suboptimal iodine nutrition among lactating mothers, emphasizing the necessity of iodine supplementation during lactation. Iodine supplementation with daily iodine fortified milk containing 150 µg iodine per cup, ensured adequate iodine status in lactating mothers, as confirmed by the median urinary and breast milk values. 

The main challenge in using median urinary iodine in infants is the difficulty faced in sample collection; hence, the best criteria for assessing iodine status have not yet been established, due to a lack of sufficient data for urinary iodine levels in this age group [8]. On the other hand, it is well-known that maternal iodine sufficiency is particularly important for exclusively breastfed infants, for whom breast milk is the sole source of iodine nutrition during a critical period of growth and development. Hence, on a biological basis, it could be expected that a positive association between urinary iodine in mothers, and exclusively breastfed infants, could be found [36]. However, our findings demonstrate that, despite decreased urinary and breast iodine levels among lactating mothers of the control group during the period of study, the iodine nutrition status of their infants was within optimal levels; moreover, the urinary iodine of the infants did not show any differences between groups, indicating that a compensatory mechanism in the mammary glands probably provides iodine enriched milk to infants. Another explanation for this finding can be attributed to the higher levels of urinary iodine in the intervention group at the baseline, which may dilute the effect of iodine fortified milk on infant urinary iodine.

Furthermore, it is worth mentioning that young children are vulnerable to excessive iodine exposure, since their thyroid gland is more susceptible to the inhibitory effect of high iodine doses than the adult gland. Thyroid disturbances, i.e., subclinical hypothyroidism and overt hypothyroidism, have been reported in studies in which newborns and infants were exposed to an excessive iodine intake [49]. For instance, in Japan and China, a transient elevation of thyrotropin was reported in some neonates born to mothers chronically exposed to high doses of iodine, mainly from foods and drinking water [50,51]. Also, in Nepal, a prevalence of 7.4% subclinical hypothyroidism and <1% overt hypothyroidism was reported among infants aged 6–24 months who were exposed to an excessive iodine intake [52]. Hence, with respect to high levels of iodine in fortified milk in the present study, caution should be taken when applying these results in regions with a high daily per capita consumption of milk.

The main strengths of this research were: An investigation of the effect of iodine fortified milk on the iodine status of lactating mothers and their infants in an area with a successful salt iodization program, which is the first of its kind; the inclusion of a control group which only received iodized salt; and several measurements of breast milk and urinary iodine concentrations at different time points during the period of supplementation. However, the limitations of the present study are that we could not assess the effects of iodine fortified milk during the entire six month exclusive breastfeeding period, for further confirmation of our results; iodine concentration was measured in single spot urine and breast milk samples, which may not reflect the true iodine status in mothers and their infants. Although thyroglobulin has been reported to be a useful biomarker in populations, thyroglobulin was not measured in our study [53,54] and we were unable to estimate dietary iodine intakes through the amount of iodized salt consumption as the main dietary source of iodine in Iran. Also, we had to assign the lactose intolerant mothers to the control group. However, the randomization and findings of the study do not seem to be affected, due to the small number of these mothers (*n* = 3).

## 5. Conclusions

Findings of the current study indicate that, in an area with a successful salt iodization program, lactating mothers require additional iodine through iodine fortified foods or iodine supplements. Our results also show that the supplementation of lactating mothers with daily iodine fortified milk containing 150 µg iodine per cup, can ensure iodine nutrition adequacy; however, iodine fortified milk had no effect on the iodine status of infants, who had iodine sufficient status due to the high iodine concentrating capacity in the mammary gland. It seems that iodine fortified milk, in addition to iodized salt, can be considered a good dietary source to ensure iodine sufficiency during the lactation period. 

## Figures and Tables

**Table 1:** Baseline characteristics of lactating mothers and their infants in the iodine fortified milk and control groups.

Characteristics	Iodine Fortified Milk (*n* = 42)	Control (*n* = 42)	*p*
*Maternal*			
Age (year)	27.7 ± 4.5	28.5 ± 4.5	0.356
Education (year)	10.8 ± 2.6	11.2 ± 3.7	0.355
Occupation (housekeeper), *n* (%)	40 (95.2)	38 (92.7)	0.625
Time ofprior pregnancy (year)	3.1 ± 3.6	3.4 ± 3.6	0.774
Gravidity, *n* (%)			0.524
Primigravidity	17 (40.5)	11 (26.8)	
Multigravidity	25 (59.5)	30 (73.2)	
Parity, *n* (%)			0.546
Primiparity	20 (47.6)	17 (41.5)	
Multiparity	22 (52.4)	24 (58.5)	
Delivery type, *n* (%)			0.690
NVD	14 (33.3)	12 (29.3)	
CS	28 (66.7)	29 (70.7)	
History of abortion, *n* (%)			0.261
Yes	7 (16.7)	11 (26.8)	
No	35 (83.3)	30 (73.2)	
Use of iodine containing supplements during pregnancy, *n* (%)			0.485
Yes	3 (7.1)	2 (4.9)	
No	29 (69.0)	33 (80.5)	
Do not know	10 (23.8)	6 (14.6)	
*Neonatal*			
Sex, *n* (%)			0.708
Male	26 (61.9)	27 (65.9)	
Female	16 (38.1)	14 (34.1)	
Birth weight (g)	3187 ± 344	3251 ± 348	0.603
Birth height (cm)	49.7 ± 1.9	50.0 ± 1.7	0.657
Birth head circumference (cm)	34.8 ± 1.2	34.5 ± 1.5	0.468
TSH (mIU/L)	1.5 ± 1.7	1.2 ± 1.1	0.350

NVD: natural vaginal delivery; CS: cesarean section; TSH: thyroid stimulating hormone.

**Table 2:** Urinary and breast milk iodine concentrations in lactating mothers and their infants in the iodine fortified milk and control groups.

Time Postpartum	Iodine Fortified Milk (*n* = 40)		Control (*n* = 40)	
	Median (IQR)	*n* (%) *	Median (IQR)	*n* (%) *
*Maternal UIC (μg/L)*				
3–5 days (baseline)	70.2 (41.2–199.0)	14 (35.9)	96.9 (47.1–193.8)	16 (45.7)
7 days	118.7 (68.0–161.7) **	23 (59.0) **	51.0 (38.2–77.8)	6 (15.8)
10 days	131.7 (85.3–188.4)	26 (74.3)	103.4 (88.6–140.7)	18 (60)
14 days	122.8 (77.2–182.9) **	23 (60.5) **	47.5 (38.4–86.8)	5 (12.2)
1 month	104.1 (57.3–160.9)	16 (55.2)	41.1 (32.1–55.9)	2 (5.1)
*BMIC (μg/L)*				
3–5 days (baseline)	176.0 (133.7–218.7) **	26 (65.0)	215.0 (168.5–315.5)	34 (81.0)
7 days	191.0 (105.0–245.0)	27 (65.9)	176.0 (140.0–286.0)	28 (71.8)
10 days	217.0 (148.7–339.0) **	29 (74.4)	162.0 (120.0–206.5)	22 (55.0)
14 days	242.0 (156.2–355.7) **	30 (78.9)	160.0 (115.2–199.2)	23 (60.5)
1 month	210.0 (100.0–286.0) **	25 (64.1)	142.0 (92.2–197.2)	19 (47.5)
*Infant UIC (μg/L)*				
3–5 days (baseline)	231.2 (91.7–268.2)	13 (72.2)	192.8 (79.4–244.6)	16 (61.5)
7 days	168.7 (86.2–326.4)	18 (75.0)	120.0 (69.7–219.3)	16 (64.0)
10 days	218.9 (138.4–293.1) **	17 (85.0)	138.3 (60.0–191.5)	16 (61.5)
14 days	194.3 (122.1–306.0) **	24 (80.0) **	115.7 (75.2–223.3)	22 (56.4)
1 month	230.2 (71.0–317.5) **	14 (73.7)	110.4 (47.2–197.0)	19 (52.8)

UIC: urinary iodine concentration; BMIC: breast milk iodine concentration; IQR: interquartile range. * Normal values: urinary iodine concentration ≥100 µg/L and breast milk iodine concentration ≥150 µg/L; ** Significantly different from control group.

**Table 3:** Effects of iodine fortified milk on maternal and infant urinary and breast milk iodine concentrations.

Variables	Ratio of Means (95% CI) *	p **
Maternal urinary iodine concentration		
Model I ^†^	1.58 (1.53–1.65)	<0.001
Model II ^‡^	1.85 (1.77–1.93)	<0.001
Breast milk iodine concentration		
Model I ^†^	1.11 (1.07–1.16)	0.096
Model II ^‡^	1.25 (1.19–1.32)	<0.001
Infant urinary iodine concentration		
Model I ^§^	1.31 (1.24–1.38)	0.021
Model II ^€^	1.19 (0.69–1.45)	0.400

CI: confidence interval. * Ratio of means (95% CI) with log-transformed data; ** Linear mixed models were used to compare two groups for overall means during the period of study; ^†^ Adjusted for mothers’ occupation, gravidity, use of iodine containing supplements during pregnancy, and iodine content of salt; ^‡^ Further adjusted for mothers’ occupation, gravidity, use of iodine containing supplements during pregnancy, iodine content of salt, and baseline values; ^§^ Adjusted for mothers’ occupation, gravidity, use of iodine containing supplements during pregnancy, iodine content of salt, birth weight, and frequency use of formula; ^€^ Further adjusted for mothers’ occupation, gravidity, use of iodine containing supplements during pregnancy, iodine content of salt, birth weight, frequency use of formula, and baseline values.
